# The role of emotion dysregulation in self-management behaviors among adults with type 2 diabetes

**DOI:** 10.1007/s10865-024-00483-5

**Published:** 2024-04-26

**Authors:** Sophie R. Kollin, Kim L. Gratz, Aaron A. Lee

**Affiliations:** 1 Department of Psychology, University of Mississippi, 304 University Circle, Oxford, 38677 MS England; 2 Lyra Health, Burlingame, CA USA; 3 Department of Psychology, University of Toledo, Toledo, OH USA

**Keywords:** Diabetes, Chronic disease, Emotion dysregulation, Disease self-management, Self-care behaviors

## Abstract

Suboptimal disease self-management among adults with type 2 diabetes is associated with greater risk of diabetes related health complications and mortality. Emotional distress has been linked with poor diabetes self-management; however, few studies have examined the role of emotion dysregulation in diabetes management. The purpose of this study was to examine the relations between different facets of emotion dysregulation and diabetes self-management behaviors among a sample of 373 adults with type 2 diabetes. Separate median regression and binary logistic regression models were used to examine the association of emotion dysregulation facets and each diabetes self-care behavior (i.e., medication nonadherence, diet, exercise, self-monitoring of blood glucose (SMBG), foot care, and smoking). Generally, greater difficulties in emotion regulation were associated with poorer self-management behaviors. However, several facets of emotion dysregulation were linked with better self-management behaviors. Addressing emotion dysregulation among adults with type 2 diabetes has the potential to improve diabetes related self-management.

Diabetes is a significant public health issue in the U.S., with approximately 1 in 10 Americans currently diagnosed with type 2 diabetes (Centers for Disease Control and Prevention, 2021). Notably, prevalence rates of diabetes in the U.S. have been projected to increase by 54% between 2015 and 2030, suggesting that type 2 diabetes will continue to be a serious health issue in the next decade (Rowley et al., 2017). Total costs of diabetes, including direct medical costs and reduced productivity costs, have increased by $82 billion dollars from 2012 to 2017 (American Diabetes Association, 2018). Moreover, adults with type 2 diabetes are at a significantly greater risk of mortality and health complications, including heart and kidney disease (Afkarian et al., 2013; Emerging Risk Factors Collaboration, 2010).

Effective self-management of type 2 diabetes can improve diabetes related health outcomes, including greater glycemic control (i.e., lower glycated hemoglobin A1c) and reduced risk of irreversible, disabling diabetes complications (Chrvala et al., 2016; Gao et al., 2013). However, self-management regimens for type 2 diabetes can be burdensome, as they include adherence to daily medication regimens, regular physical activity, following a diet plan, and regular self-monitoring of blood glucose levels (American Diabetes Association, 2015). Evidence suggests that, on average, adults with type 2 diabetes have suboptimal self-management behaviors (Nicolucci et al., 2013). For instance, among adults with type 2 diabetes, approximately 38% do not engage in daily exercise or foot care, around 55% do not follow dietary guidelines, and more than one third are nonadherent to their diabetes medications (Iglay et al., 2015; Safford et al., 2005). A recent systematic review found that adults with diabetes are adherent to recommendations for diet an average of 3.5 days per week, exercise an average of 3 days per week, self-monitoring of blood glucose 1 day per week, and foot care an average of 3 days per week (Mogre et al., 2019). Indeed, most adults with type 2 diabetes do not meet clinical targets for cardiometabolic control. For example, a large national survey found that only 18.8% of U.S. adults with diabetes achieved recommended targets for A1c, blood pressure, and LDL cholesterol (A1c < 7.0%, BP < 130/80 mmHg, LDL < 100 mg/dl; Casagrande et al., 2013). Together, these findings indicated that many adults with diabetes have difficulty effectively managing their diabetes. Unfortunately, poor diabetes self-management and glycemic control are associated with greater risk of disabling diabetes related complications and mortality (Currie et al., 2012; Feldman et al., 2014; Ho et al., 2006; Zulman et al., 2012).

Given the serious consequences of poor diabetes self-management, it is important to understand factors that may contribute to suboptimal self-care behaviors. Diabetes self-management regimens are demanding and a common source of significant emotional distress (Fisher et al., 2010; Polonsky et al., 2005). Difficulties regulating emotions may thus undermine patients’ abilities to effectively navigate complex and onerous self-management routines (Wierenga et al., 2017). Emotion dysregulation is characterized by difficulties understanding, accepting, and responding effectively to emotions (Gratz & Roemer, 2004). Current conceptualizations of emotion dysregulation suggest that this is a multifaceted construct, encompassing difficulties with the awareness, understanding, and acceptance of emotions, difficulties controlling behaviors when distressed (including controlling impulsive behaviors and engaging in goal-directed behavior), and difficulties accessing effective emotion regulation strategies (Gratz & Roemer, 2004). A recent integrative review found that adults with a chronic illness are more likely to have difficulties regulating their emotions (Wierenga et al., 2017). Moreover, among adults with chronic illness (e.g., Multiple Sclerosis, Rheumatoid Arthritis, Obesity, Coronary Heart Disease), difficulties with emotion regulation are associated with lower quality of life, worse psychosocial outcomes, and poorer health indicators (Gianini et al., 2013; Karademas et al., 2011; Phillips et al., 2009; Van Middendorp et al., 2005).

Among adults with type 2 diabetes, maladaptive emotion regulation strategies (e.g., self-blame, rumination, and catastrophizing) are linked to greater diabetes related distress and physical symptom burden (Kane et al., 2018). Relatedly, a small body of research has found that poor cognitive affective regulation is associated with higher diabetes distress, which is linked to worse self-management (Coccaro et al., 2022; Fisher et al., 2018). Positive emotional coping is associated with better diabetes self-management behaviors, suggesting that adaptive emotion regulation may play an important role in positive health outcomes (Smalls et al., 2012). One prior study of adults with type 2 diabetes found no significant differences in emotion regulation between those with high versus controlled HbA1c levels (Rasmussen et al., 2013). However, this study had two important limitations that warrant consideration. First, as the authors note, the study did not examine the relations between unique facets of emotion regulation difficulties and glycemic control. Second, this study did not examine the relations between emotion regulation difficulties and critical diabetes self-management behaviors (e.g., diet, exercise, self-monitoring of blood glucose, medication adherence) that may be directly impacted by patients’ difficulties regulating negative emotions. Indeed, no studies to date have examined the relations between facets of emotion dysregulation and self-management behaviors among adults with type 2 diabetes. Given existing research demonstrating that targeted interventions are effective at improving deficits in emotion regulation (Gratz et al., 2014; Neacsiu et al., 2014), identification of links between specific emotion regulation difficulties and self-management behaviors has the potential to inform the development of programs to improve diabetes self-management among adults with type 2 diabetes.

The current study aimed to address this gap in the literature by examining the association between facets of emotion dysregulation and specific self-management behaviors (i.e., medication nonadherence, diet, exercise, self-monitoring of blood glucose (SMBG), foot care, and smoking) among adults with type 2 diabetes. We hypothesized that greater difficulties regulating emotions would be significantly associated with worse self-management, adjusting for important patient characteristics related to self-care behaviors (i.e., insulin use, age, diabetes duration, insurance status).

## Method

**Participants and procedures**.

Participants were recruited via a web-based panel of adults with diabetes maintained by CloudResearch. To be included, participants had to be 18 years of age or older and endorse a current diagnosis of type 2 diabetes. Those who endorsed a diagnosis of type 1 diabetes were excluded from participation. Participants were asked to provide informed consent and then complete a battery of questionnaires. 518 individuals provided informed consent for the study and of those individuals, 428 reported a diagnosis of diabetes. Of those participants, 40 started but did not complete the survey, 14 indicated not having a diagnosis of type 2 diabetes, and one individual did not pass the attention checks. The final sample of participants that completed the survey included 373 adults with type 2 diabetes.

### Measures

#### Difficulties in emotion regulation

The Difficulties in Emotion Regulation Scale (DERS) was used to measure emotion dysregulation (Gratz & Roemer, 2004). The DERS is comprised of 36 items rated on a Likert-type scale from 1 (“Almost Never”) to 5 (“Almost Always”). Total scores on the DERS range from 36 to 180, with higher scores indicating greater difficulties in emotion regulation. The DERS has six subscales: nonacceptance of negative emotions (Nonacceptance; e.g., “When I’m upset, I feel guilty for feeling that way”), difficulties engaging in goal-directed behaviors when distressed (Goals; e.g., “When I’m upset, I have difficulty getting work done”), difficulties controlling impulsive behaviors when distressed (Impulsivity; e.g., “When I’m upset, I become out of control”), lack of emotional awareness (Awareness; e.g., “I pay attention to how I feel”), difficulties accessing effective emotion regulation strategies (Strategies; e.g., “When I’m upset, I believe that there is nothing I can do to make myself feel better”), and lack of emotional clarity (Clarity; e.g., “I know exactly how I am feeling”). The DERS has shown good internal consistency (α = .93) and test-retest reliability, as well as adequate construct and predictive validity (Gratz & Roemer, 2004). The total scale had excellent internal consistency in the present study (α = .96). Internal consistency for the subscales ranged from acceptable to excellent (clarity: α = .74; awareness: α = .79; goals: α = .86; impulsivity: α = .90; strategies: α = .93; nonacceptance: α = .94).

#### Diabetes self-management behaviors

The Summary of Diabetes Self-Care Activities measure (SDSCA) was used to measure participants’ diabetes self-management behaviors (Toobert et al., 2000). Participants rated their adherence to diet (e.g., “How many of the last 7 days did you eat five or more servings of fruit and vegetables?”), exercise (e.g., “How many of the last 7 days did you participate in at least 30 minutes of physical activity?”), blood sugar testing (e.g., “How many of the last 7 days did you test your blood sugar?”), and foot care (e.g., “How many of the last seven days did you check your feet?”) over the past week from 0 days to 7 days. Participants also indicated if they had smoked a cigarette over the past 7 days (0 = No, 1 = Yes). Correlations between the items in each subscale ranged from moderate to strong in the present study (Diet: *rho* = 0.80; Exercise: *rho* = 0.82; Blood sugar testing: *rho* = 0.75; Foot care: *rho* = 0.50). Medication nonadherence was measured using 3 questions from the Extent of and Reasons for Nonadherence Scale (Voils et al., 2012). Participants rated their adherence to prescribed diabetes medications (e.g., “Over the past 7 days, I took all doses of my diabetes medications”) during the past week on a Likert-type scale from 1 (“Never”) to 5 (“Always”). This scale had acceptable internal consistency in the present study (α = 0.72).

#### Control variables

Age, health insurance status, insulin use, and diabetes duration were collected via self-report. Participants were asked if they had health insurance in the past 12 months (0 = no insurance, 1 = insurance) and if they were currently prescribed insulin to help manage their diabetes (0 = no insulin use, 1 = insulin use). Diabetes duration (years) was calculated by subtracting participants self-reported age at diagnosis from their age at the time of data collection.

### Data analysis

All analyses were performed using SPSS version 28.0. Descriptive statistics were used to characterize the sample. Univariate distributions for each diabetes self-care behavior were examined to assess normality and identify outliers. The distributions for weekly frequency of each self-care behavior were largely non-normal, suggesting that the mean was not representative of central tendency. Consequently, median regression was used to evaluate the association of emotion dysregulation facets and each self-care behavior at the 50th percentile. Due to the high intercorrelation between subscales (Online Resource 1), separate median regression models were used to examine the association of each DERS subscale with each diabetes self-care behavior (i.e., nonadherence, exercise, general diet, self-monitoring of blood glucose, foot care). Binary logistic regression models with each DERS subscale for smoking behavior (current non-smoker = 0, current smoker = 1) were also conducted. All regression models included established predictors of self-care behaviors including age, health insurance status, diabetes duration, and insulin use (Gonzalez-Zacarias et al., 2016; Johnson et al., 2014; Ogbera & Adeyemi-Doro, 2011). All tests were two-tailed. In order to control the probability of Type I error, a false discovery rate adjusted critical *p* value was used (Benjamini & Hochberg, 1995). We derived an overall critical significance level of *p* < .009 using a maximum acceptable false discovery rate of 0.027 for all reported tests (*n* = 36, i.e., one acceptable false positive for all 36 tests).

## Results

### Sample characteristics

Sociodemographic and clinical characteristics of the sample (*N* = 373) are reported in Table 1. The sample was primarily White, non-Hispanic, and female with an average age of approximately 55 years (range: 19 to 85). Most participants had a high school degree, and the modal annual household income was greater than $75,000. Most of the sample had health insurance and a primary care provider during the prior 12 months. Participants had an average diabetes duration of roughly 12 years, and approximately 39% of the sample was using insulin at the time of data collection.

**Table 1:** Participant characteristics (*N* = 373)

Variable	M (SD) or n (%)
Age (in years)	54.9 (15.67)
Female Sex	212 (56.8%)
*Race*	
White	293 (78.6%)
Black	47 (12.6%)
Asian	13 (3.5%)
American Indian	4 (1.1%)
Other	16 (4.2%)
*Ethnicity*	
Hispanic/Latino	37 (9.9%)
*Education*	
8th grade or less	1 (0.3%)
Some high school, but did not graduate	7 (1.9%)
High school graduate or GED	75 (20.1%)
Some college or 2-year college degree	131 (35.1%)
4-year college graduate	95 (25.5%)
More than 4-year college degree	64 (17.2%)
*Current income*	
< $15,000	35 (9.4%)
$15,000–30,000	75 (20.1%)
$30,000–50,000	74 (19.8%)
$50,000–75,000	78 (20.9%)
>$75,000	111 (29.8%)
Insulin Use	144 (38.6%)
Diabetes Duration (in years)	12.6 (10.15)
Primary Care Provider	362 (97.1%)
Health Insurance	355 (95.2%)
DERS Total Score	83.7 (28.05)

### Medication nonadherence

Results of median regression models examining predictors of medication nonadherence are reported in Table 2. Greater difficulties in the facets of emotion dysregulation involving the nonacceptance of emotions (*B* = 0.05, *SE* = 0.01, *p* < .001), difficulties engaging in goal directed behaviors when distressed (*B* = 0.05, *SE* = 0.01, *p* < .001), difficulties controlling impulsive behaviors when distressed (*B* = 0.09, *SE* = 0.01, *p* < .001), lack of emotional clarity (*B* = 0.10, *SE* = 0.02, *p* < .001), and difficulties accessing effective emotion regulation strategies (*B* = 0.05, *SE* = 0.01, *p* < .001) were significantly associated with greater medication nonadherence. However, difficulties with emotional awareness (*B* < 0.001, *SE* = 0.01, *p* = 1.00) were not significantly associated with medication nonadherence.

**Table 2:** Median regression models examining the predictors of medication nonadherence

Variables	B	SE	95% CI	
*Control*				
Insulin use	0.17	0.12	-0.06	0.42
Diabetes duration	0.01	0.01	-0.01	0.02
Age	-0.03	0.004	-0.03	-0.02
Insurance status	-0.16	0.30	-0.75	0.43
*DERS subscales*				
Nonacceptance	**0.05**	0.01	0.03	0.07
Goals	**0.05**	0.01	0.02	0.07
Impulse	**0.09**	0.01	0.07	0.11
Awareness	< 0.001	0.01	-0.03	0.03
Clarity	**0.10**	0.02	0.06	0.13
Strategies	**0.05**	0.01	0.03	0.06

*B* = Unstandardized Regression Coefficient

Bold typeface indicates significant association using the false discovery corrected *p* value (*p* < .009)

### Exercise

Results of median regression models examining predictors of diabetes self-care behaviors, including exercise, general diet, SMBG, and foot care are reported in Table 3. Greater nonacceptance of emotions (*B* = 0.10, *SE* = 0.03, *p* = .001) and greater difficulties controlling impulsive behaviors when distressed (*B* = 0.14, *SE* = 0.04, *p* < .001) were associated with significantly more exercise. Greater difficulties with emotional awareness (*B* = -0.21, *SE* = 0.04, *p* < .001) were associated with significantly less exercise. Difficulties engaging in goal directed behaviors when distressed (*B* = 0.06, *SE* = 0.04, *p* = .184), lack of emotional clarity (*B* = -0.0003, *SE* = 0.06, *p* = .958), and difficulties accessing effective emotion regulation strategies (*B* = 0.05, *SE* = 0.03, *p* = .043) were not significantly associated with exercise.

**Table 3:** Median regression models examining the predictors of diabetes self-care behaviors

Variables	Exercise				General Diet				SMBG				Foot Care			
Controls	*B*	*SE*	95%	CI	*B*	*SE*	95%	CI	*B*	*SE*	95%	CI	*B*	*SE*	95%	CI
Insulin Use	0.14	0.41	-0.68	0.95	< 0.001	0.28	-0.56	0.56	1.63	0.43	0.79	2.47	-0.05	0.41	-0.86	0.76
Diabetes Duration	-0.03	0.02	-0.07	-0.01	< 0.001	0.01	-0.03	0.03	0.01	0.02	-0.04	0.05	0.02	0.02	-0.02	0.06
Age	-0.07	0.01	-0.10	-0.04	< 0.001	0.01	-0.02	0.02	0.02	0.01	-0.01	0.05	-0.02	0.01	-0.05	0.01
Insurance Status	0.17	0.89	-1.58	1.93	0.50	0.61	-0.71	1.71	2.06	0.97	0.15	3.97	0.34	0.89	-1.41	2.09
*DERS subscales*																
Nonacceptance	**0.10**	0.03	0.04	0.16	0.05	0.02	0.01	0.09	0.07	0.03	-0.003	0.13	0.08	0.03	0.02	0.14
Goals	0.06	0.04	-0.03	0.14	< 0.001	0.03	-0.06	0.06	0.01	0.05	-0.08	0.10	0.003	0.04	-0.08	0.09
Impulse	**0.14**	0.04	0.07	0.21	0.04	0.03	-0.01	0.09	0.04	0.04	-0.04	0.12	0.02	0.04	-0.05	0.10
Awareness	**-0.21**	0.04	-0.27	-0.14	**-0.16**	0.02	-0.21	-0.12	**-0.24**	0.04	-0.32	-0.17	**-0.17**	0.04	-0.25	-0.09
Clarity	-0.003	0.06	-0.12	-0.11	< 0.001	0.04	-0.08	0.08	-0.15	0.06	-0.27	-0.02	-0.02	0.05	-0.13	0.09
Strategies	0.05	0.03	0.002	0.10	< 0.001	0.02	-0.04	0.04	-0.002	0.03	-0.06	0.06	0.01	0.03	-0.05	0.06

*B* = Unstandardized Regression Coefficient, SMBG = Self-Monitoring of Blood Glucose

Bold typeface indicates significant association using the false discovery corrected *p* value (*p* < .009)

### General diet

Greater difficulties with emotional awareness (*B* = -0.16, *SE* = 0.02, *p* < .001) were associated with significantly worse general diet. However, difficulties involving emotional nonacceptance (*B* = 0.05, *SE* = 0.02, *p* = .009), engaging in goal directed behaviors when distressed (*B* < 0.001, *SE* = 0.03, *p* = 1.00), controlling impulsive behaviors when distressed (*B* = 0.04, *SE* = 0.03, *p* = .331), lack of emotional clarity (*B <* 0.001, *SE* = 0.04, *p* = 1.00), and accessing effective emotion regulation strategies (*B <* 0.001, *SE* = 0.02, *p* = 1.00) were not significantly associated with general diet.

### Self-monitoring of blood glucose

Greater difficulties with emotional awareness were associated with significantly worse self-monitoring of blood glucose (*B* = -0.24, *SE* = 0.04, *p* < .001). However, nonacceptance of emotions (*B* = 0.07, *SE* = 0.03, *p* = .061), difficulties with goal directed behavior (*B* = 0.01, *SE* = 0.05, *p* = .772), difficulties controlling impulsive behaviors (*B* = 0.04, *SE* = 0.04, *p* = .359), lack of emotional clarity (*B* = -0.15, *SE* = 0.06, *p* = .020), and difficulties accessing effective emotion regulation strategies (*B* = -0.002, *SE* = 0.03, *p* = .951) were not significantly associated with self-monitoring of blood glucose.

### Foot care

Greater difficulties with emotional awareness were associated with significantly worse foot care (*B* = -0.17, *SE* = 0.04, *p* < .001). However, nonacceptance of emotions (*B* = 0.08, *SE* = 0.03, *p* = .015), difficulties with goal directed behavior (*B* = 0.003, *SE* = 0.04, *p* = .939), difficulties controlling impulsive behaviors (*B* = 0.02, *SE* = 0.04, *p* = .504), lack of emotional clarity (*B* = -0.02, *SE* = 0.05, *p* = .696), and difficulties accessing effective emotion regulation strategies (*B* = 0.01, *SE* = 0.03, *p* = .812) were not significantly associated with foot care.

### Smoking

Results of binary logistic regression models examining predictors of smoking are reported in Table 4. None of the facets of emotion dysregulation examined here were significantly associated with smoking (*ps* > 0.009).

**Table 4:** Binary logistic regression models examining the predictors of smoking

Controls	B	SE	95% CI	
Insulin Use	-0.17	0.26	0.51	1.40
Diabetes Duration	-0.01	0.01	0.96	1.01
Age	-0.04	0.01	0.94	0.98
Insurance Status	-0.65	0.50	0.20	1.39
*DERS subscales*				
Nonacceptance	0.01	0.02	0.97	1.05
Goals	0.02	0.03	0.97	1.07
Impulse	0.03	0.02	0.99	1.08
Awareness	0.001	0.03	0.95	1.05
Clarity	0.02	0.04	0.95	1.09
Strategies	0.02	0.02	0.99	1.06

*B* = Unstandardized Regression Coefficient

## Discussion

Prior research provides evidence for associations between difficulties in emotion regulation and poor health outcomes among individuals with chronic illness (Gianini et al., 2013; Karademas et al., 2011; Phillips et al., 2009; Van Middendorp et al., 2005). Among adults with diabetes, emotion dysregulation has been found to be associated with greater diabetes related distress, which, in turn, has been linked to worse disease self-management (Coccaro et al., 2022; Fisher et al., 2018). This study is the first to examine the associations between facets of emotion dysregulation and specific diabetes self-management behaviors. Findings from the current study provide partial support for study hypotheses, suggesting that greater difficulties with emotion regulation in some domains are associated with worse self-management behaviors.

### Medication nonadherence

We found that all emotion dysregulation facets, with the exception of difficulties with emotional awareness, were associated with greater medication nonadherence. These results suggest that emotion dysregulation plays a particularly important role in medication-taking behaviors among adults with diabetes. Adherence to daily medication regimens is a highly routinized task that can be burdensome for adults with diabetes. Unlike other diabetes self-management behaviors, taking medications involves adherence to specific treatment regimens and, thus, requires significant behavioral and cognitive control, including memory, planning, and implementation (e.g., Whitley et al., 2006). Given the complexity of diabetes medication regimens and the cognitive and behavioral resources necessary to successfully adhere to these regimens, this self-management behavior may be especially likely to be negatively influenced by difficulties regulating emotions. Consistent with the current findings, past research has found that individuals who experience greater difficulties processing their emotions are more likely to be nonadherent to diabetes medication and diabetes self-management regimens (Hughes et al., 2012; Smalls et al., 2012). Further, emotion dysregulation, as measured by the DERS, has been associated with poorer acne medication adherence and poorer medication adherence among healthy emerging adults (Singh & Singh, 2023; Turan et al., 2020). These findings suggest that emotion dysregulation may interfere with an individual’s ability to engage in self-care activities that rely on consistent cognitive and behavioral control, such as taking daily diabetes medications.

Alternatively, the exacerbation of distress that accompanies maladaptive responses to emotions may help explain the relation of multiple emotion dysregulation facets to diabetes medication nonadherence. For example, nonaccepting responses to emotional distress, difficulties understanding and labeling emotions, and lack of access to effective emotion regulation strategies have been found to increase distress and emotional suffering, which, in turn, may make medication adherence more challenging. This is a particularly relevant consideration given evidence that adults with diabetes experience significant emotional distress related to their diabetes (Polonsky et al., 2005). Consistent with this suggestion, past research has found that emotional distress resulting from diabetes is associated with worse medication adherence (Aikens, 2012; Jannoo et al., 2017; Kretchy et al., 2020; Zhang et al., 2021). Further, several studies have demonstrated that emotion dysregulation is related to greater diabetes distress, which, in turn, is associated with worse diabetes self-care (Coccaro et al., 2022; Fisher et al., 2018). Together, these findings suggest that exacerbation of diabetes-related emotional distress due to maladaptive responses to emotions may make medication adherence more challenging.

### Diabetes self-care behaviors

Difficulties with emotional awareness emerged as a particularly important facet of emotion dysregulation for diabetes self-management. We found that greater difficulties with emotional awareness were associated with *less* exercise, *worse* general diet, *worse* SMBG, and *worse* foot care. Prior research investigating similar emotion regulation difficulties has examined poor emotional processing or difficulties exploring and acknowledging emotions. Consistent with the current findings, this research found that poor emotional processing was associated with worse general diet, less exercise, and worse SMBG on the SDSCA (Smalls et al., 2012). Taken together, these findings suggest that emotional awareness may be particularly important for managing type 2 diabetes, likely due to the functional nature of emotions and the information they provide. Specifically, individuals who attend to their emotions (vs. avoiding or ignoring their emotions) are more likely to receive important information from their emotions about themselves and their environment – information that can guide the individual’s behavior and inform more effective decision making. It is possible that adults with type 2 diabetes who experience greater difficulties with emotional awareness may be more likely to suppress or ignore the important information being provided by their emotions, including awareness of bodily sensations that could prompt self-management behaviors. Indeed, previous literature supports this suggestion, revealing a link between emotional awareness and somatic awareness (Kanbara & Fukunaga, 2016; Wiens et al., 2000).

Contrary to our hypothesis, we found that greater nonacceptance of emotions and greater difficulties controlling impulsive behaviors when distressed were associated with more exercise among adults with type 2 diabetes. Although unexpected, it is possible that these findings reflect the potential for exercise to be used as an avoidant emotion regulation strategy. That is, although exercise in general is considered an effective diabetes self-management behavior, it can also function to avoid or escape emotional distress, especially if done to the extreme. For instance, research has found that among both clinical and nonclinical samples, exercise is used as a strategy to regulate negative emotions (Bratland-Sanda et al., 2010; De Young & Anderson, 2010; Edwards et al., 2017). Exercise may also be a compensatory behavior to reduce distress caused by secondary emotional responses to emotions, such as shame or guilt. Consistent with this suggestion and current findings, greater nonacceptance of emotions, as measured by the DERS, uniquely predicted greater exercise among women with eating disorder psychopathology (McClure et al., 2022). Thus, it may be that among adults with type 2 diabetes in the current study, greater emotional nonacceptance and difficulties controlling impulsive behaviors when distressed prompted increased exercise in an effort to regulate or avoid emotional distress (resulting in a positive association between these facets of emotion dysregulation and exercise).

### Limitations

The current findings should be considered in the context of several important limitations. First, this observational study employed a cross-sectional design. Therefore, we were unable to determine the directionality of the relations between different facets of emotion regulation and diabetes self-care behaviors. For instance, it is unclear whether difficulties in emotion regulation result in poorer self-management behaviors or whether poorer self-management behaviors contribute to difficulties in emotion regulation. Nevertheless, the present findings suggest a link between some facets of emotion regulation and specific diabetes self-care behaviors. Second, this study used a validated self-report measure of diabetes self-care activities that relied on participant’s retrospective self-report of these behaviors and, therefore, may be influenced by recall bias. Future research may benefit from using ecological momentary assessment of self-care behaviors or electronic monitoring of patient’s self-management behaviors (e.g., use of glucometers, pharmacy refill records). Additionally, our analyses did not account for participants’ overall negative affect or frequency or intensity of negative emotional experiences, which may be important to consider when examining the relation of emotion regulation difficulties to diabetes self-management behaviors. For example, difficulties regulating negative emotions may be less relevant to self-management behaviors among individuals who experience negative emotions less often or less intensely. Finally, the current study utilized online data collection methods, and therefore study participants were not assessed in person. However, to ensure the reliability of the data, participants were required to pass two separate attention checks.

## Conclusions

The current study addresses an important gap in the literature by examining the relationship between different facets of emotion regulation and diabetes self-care behaviors. Our results suggest that emotion regulation difficulties in general appear to be especially relevant to medication nonadherence, with multiple dimensions of emotion regulation difficulties associated with greater medication nonadherence. In addition, difficulties with emotional awareness appear to play a particularly important role in other diabetes self-care activities, as this facet of emotion regulation was associated with *less* exercise, *worse* general diet, *worse* SMBG, and *worse* foot care. Prior research has found a negative association between difficulties with emotional awareness and somatic awareness (Kanbara & Fukunaga, 2016; Wiens et al., 2000). Given that diabetes self-care activities require individuals to respond to physical symptoms, an awareness of one’s emotional and bodily sensations, including signs of changes in blood sugar levels, is particularly important.

The current results have the potential to inform the development of programs that target emotion regulation to improve diabetes self-management. For instance, interventions designed to target emotional awareness may result in greater somatic awareness among those with diabetes, leading to more positive self-management behaviors. A pilot, randomized clinical trial found that an emotion regulation behavioral intervention resulted in improvements in A1c levels and reductions in diabetes distress, suggesting that interventions targeting emotion regulation could potentially reduce the emotional distress associated with managing diabetes and improve glucose management (Coccaro et al., 2022). Although preliminary, the current findings suggest that targeting emotional awareness may be a beneficial strategy for improving health among adults with type 2 diabetes.

## Data Availability

Data and code from the current study will be made available to individuals upon request.
